# Austin County Medical Society

**Published:** 1904-08

**Authors:** Otto E. Steck

**Affiliations:** Sec.-Treas.


Austin County Medical Society.—The Austin County Medical Society (which was organized on December 30, 1903, in Bellville by Dr. Jno. T. Moore) met at Wallis, Texas, on June 28th at 8 p. m. Dr. W. T. Brown of Wallis read a paper on "Tr. Veratrum Veride in Puerperal Convulsions," and Dr. B. E. Knolle of Industry read one on "Malarial Hematuria." Both papers were well presented and freely discussed.

On the program for the next meeting, which will be held at Sealy, July 28th, at 8 p. m., are Drs. J. S. Davidson, San Felipe; A. W. Thompson, Bellville; A. J. Knolle, Industry, and J. W. Leonard, Peters. We have a membership of fifteen.

Otto E. Steck, Sec.-Treas.

